# Interobserver Agreement in the Diagnosis of Inflammatory Bowel Disease-Associated Neoplasia in China in Comparison to Subspecialized American Gastrointestinal Pathologists

**DOI:** 10.1155/2018/8715263

**Published:** 2018-04-23

**Authors:** Xian-rui Wu, Hua-shan Liu, Xue-ying Shi, Wei-xun Zhou, Zhi-nong Jiang, Yan Huang, Dipti M. Karamchandani, John R. Goldblum, Shu-yuan Xiao, Hong-fa Zhu, Michael M. Feely, Amy L. Collinsworth, Ashwini Esnakula, Hao Xie, Bo Shen, Ping Lan, Xiu-li Liu

**Affiliations:** ^1^Department of Colorectal Surgery, The Sixth Affiliated Hospital, Sun Yat-sen University, Guangzhou, Guangdong, China; ^2^Department of Pathology, Peking University Third Hospital, Beijing, China; ^3^Department of Pathology, Peking Union Medical College Hospital, Beijing, China; ^4^Department of Pathology, Sir Run Run Shaw Hospital, Zhejiang University School of Medicine, Hangzhou, Zhejiang, China; ^5^Department of Pathology, The Sixth Affiliated Hospital, Sun Yat-sen University, Guangzhou, Guangdong, China; ^6^Department of Pathology, Penn State College of Medicine, Hershey, PA, USA; ^7^Department of Pathology, Cleveland Clinic, Cleveland, OH, USA; ^8^Department of Pathology, The University of Chicago, Chicago, IL, USA; ^9^Department of Pathology, The Mount Sinai Hospital, New York, NY, USA; ^10^Department of Pathology and Laboratory Medicine, University of Florida, Gainesville, FL, USA; ^11^Department of Internal Medicine, Yale University School of Medicine, New Haven, CT, USA; ^12^Department of Gastroenterology/Hepatology, Cleveland Clinic, Cleveland, OH, USA

## Abstract

**Background:**

The aim of this study was to evaluate the interobserver variability in diagnosing inflammatory bowel disease (IBD)-associated neoplasia among practicing pathologists from China using telepathology, a practice of remote diagnostic consultation increasingly used nationally and internationally, and its comparison with the interpretation of subspecialized gastrointestinal (GI) pathologists from the United States (US).

**Methods:**

Eight GI pathologists from the US and 4 pathologists from China with an interest in GI pathology participated in this study. A total of 50 colonic biopsies from patients with a clinical history of IBD from 8 medical centers in China were included. All microscopic slides in each case were digitized using an Aperio system. One pathologist (XL) reviewed the digitized full-slide images, and selected areas of interest were captured at low, medium, and high magnifications at a resolution of 1712 × 1072 pixels and saved as tagged image file format (TIFF) files on read-only DVD. Each pathologist evaluated the images and selected the most appropriate diagnostic category for each case (negative, indefinite, low-grade dysplasia [LGD], high-grade dysplasia [HGD], and carcinoma). A Fleiss' kappa coefficient (*K*) analysis was performed to determine interobserver agreement and the agreement of each pathologist from China with the consensus diagnosis (defined as diagnostic agreement by at least 4 participating US GI pathologists).

**Results:**

There was substantial interobserver agreement among 4 pathologists from China on the interpretation of IBD-associated neoplasia (kappa value 0.68, 95% confidence interval: 0.56–0.78). A consensus diagnosis included negative (*n* = 22), LGD (*n* = 22), HGD (*n* = 3), carcinoma (*n* = 2), and indefinite for dysplasia (*n* = 1). Using consensus diagnoses as references, the agreement between each pathologist from China and the consensus diagnosis was substantial with kappa values ranging from 0.75 to 0.80.

**Conclusions:**

This study reveals substantial interobserver agreement for the interpretation of colonic neoplasia in IBD using digitized images among Chinese pathologists as well as between each Chinese pathologist and a consensus diagnosis generated by US GI pathologists.

## 1. Introduction

Recent studies have shown that the incidence of inflammatory bowel disease (IBD), both ulcerative colitis (UC) and Crohn's disease (CD), is increasing in Asia including China. The incidence of UC in Asia is estimated to be 0.4 to 2.1 per 100,000 versus 6 to 20.3 per 100,000 in North America and Northern Europe. The prevalence rate in Asia is 6 to 30 per 100,000 versus 21.4 to 243 per 100,000 populations in North America and Northern Europe [1]. The incidence of CD in the Asia-Pacific region is about 1.37 per 100,000 [2]. Colorectal adenocarcinoma (CAC) risk in IBD has not been well established in Asia, probably due to relatively short follow-up. Nevertheless, the cumulative risk of CAC in Asian UC patients appears to be comparable to that of the West [3, 4]. The incidence of colitis-associated CAC will likely increase in the near future in Asia including China with significant health and financial impact because of the large population.

Given the above data, prevention of CAC via colonoscopic surveillance will be increasingly needed in Asia. Interpretation of colonic biopsies in IBD patients enrolled in a surveillance program will become common in China where pathology is still practiced in a general non-subspecialized fashion in most medical centers and hospitals. Only a few large medical centers have started subspecialty pathology practices including gastrointestinal (GI) pathology. Although there are significant changes in the 2015 Surveillance for Colorectal Endoscopic Neoplasia Detection and Management in Inflammatory Bowel Disease Patients: International Consensus Recommendations (SCENIC) statement, the histologic diagnosis of dysplasia in surveillance colonic biopsies still remains critical in determining the clinical follow-up [5]. Although dysplasia of the colorectum is simply defined as an unequivocal neoplastic alteration of the epithelium that remains confined within the basement membrane of the glands within which it originated [6], it is a known diagnostic challenge to pathologists, including experienced fellowship-trained GI pathologists. Riddell et al. [6] proposed a schema for the evaluation of colonic epithelial changes in IBD which included three major categories: negative, indefinite, and positive for dysplasia with “positive for dysplasia” further divided into low and high grade.

No studies on intra- and interobserver agreement of colitis-associated dysplasia have been published from China, although several studies from the United Stated (US) and the United Kingdom (UK) have been published [6–10]. The unifying conclusion from most of these studies is that there is only fair overall interobserver agreement on IBD neoplasia interpretation of biopsy specimens, with kappa value between 0.30 and 0.40 [7–9] with the lower and upper spectrums of changes, that is, the categories of negative for dysplasia and high-grade dysplasia (HGD), having the highest interobserver agreement while indefinite for dysplasia and low-grade dysplasia (LGD) suffer from low interobserver agreement [7–9, 11]. A more recent study has shown an excellent degree of histopathological interobserver agreement for diagnosing IBD-associated neoplasia [11]. Despite the difficulties, the interpretation and grading of IBD-associated neoplasia continue to be an essential part of the clinical management of IBD patients [6, 10, 12].

Telepathology is the practice of remote diagnostic consultation of either electronically transmitted, static, digitized images, or real-time pictures obtained with the use of remote robotic microscopes [13, 14]. Intra- and interobserver agreement studies on colitis-associated dysplasia using telepathology, either through static images or a dynamic method, were published previously [7, 8]. A fair degree of interobserver agreement on IBD neoplasia (kappa = 0.4) using telepathology with static images was reported [8], while slightly lower values (kappa = 0.43) were obtained using microscopic slides [8]. A poor degree of interobserver agreement on IBD neoplasia was reported using dynamic telepathology (kappa = 0.32) which is comparable (kappa = 0.35) to using microscopic slides [7].

The aim of this study was to (1) evaluate interobserver agreement for diagnosing neoplasia in IBD surveillance colonic biopsies with telepathology among 4 Chinese pathologists using a consensus diagnosis generated by a group of 8 GI pathologists from US and (2) identify histologic features associated with grading neoplasia in IBD surveillance colonic biopsies.

## 2. Methods

### 2.1. Analysis of Captured Images from Digitized Full Slides

This study used a cohort of IBD patients from China who underwent surveillance colonoscopy with biopsies or underwent colectomy for colorectal neoplasia from 1999 to 2016 from 8 medical centers throughout China. All slides from this cohort including colonic biopsies or colorectal resection specimens were de-identified and scanned using an Aperio system (Leica Biosystems) at 20x magnification. The images were hosted in Guangzhou, Guangdong Province, China. One US GI pathologist (XL) was given internet access to all images for the entire study cohort, reviewed the full-slide images, and served as the reference pathologist. The reference pathologist selected a total of 50 colonic biopsies from this IBD cohort. In these 50 colon biopsies, the histologic areas of interest were captured at low, medium, and high magnifications at a resolution of 1712 × 1072 pixels and saved as tagged image file format (TIFF) files on a read-only DVD. A total of 3 to 6 images were captured for each case. Three months later, the images were sent on a read-only DVD to the 7 other participating GI pathologists in the US and 4 pathologists in China with an interest in GI pathology for review. A GI pathologist in the US was defined as a GI fellowship-trained pathologist practicing in an academic center and/or a pathologist with at least more than 10 years' experience of subspecialized GI sign-out. Each participating pathologist evaluated the images and selected the most appropriate diagnostic category for each case (negative, indefinite for dysplasia, LGD, HGD, and carcinoma) using previously published criteria [6]. In addition, the four participating pathologists in China were asked to assess surface maturation, nuclear enlargement, nuclear hyperchromasia, nuclear stratification, nuclear pleomorphism, loss of nuclear polarity, architectural complex, and abnormal mitoses, features which have been reported in the diagnosis of dysplasia (Table 1) [6]. All results were sent to the reference pathologist (XL) for statistical analysis. The reference pathologist (XL) finished reviewing the images prior to receiving any results from the other participating pathologists to avoid bias.

## 3. Statistics

A Fleiss' kappa coefficient (*K*) analysis was performed to determine interobserver agreement and the agreement of each pathologist from China with the consensus diagnosis. Kappa measures agreement beyond which is expected by chance alone. A kappa value < 0, 0.01–0.20, 0.21–0.40, 0.41–0.60, 0.61–0.80, and 0.81–1.0 is considered poor, slight, fair, moderate, substantial, and almost perfect agreement, respectively [15]. In addition, the sensitivity, specificity, positive predictive value, and negative predictive value of each evaluated histologic feature to diagnose IBD-associated neoplasia were calculated. R version 3.3.2 (Vienna, Austria, 2016) was used for statistical analysis. *P* value < 0.05 was the criterion for statistical significance.

## 4. Results

Images from 50 biopsies were independently reviewed by 8 GI pathologists from the US. A consensus diagnosis was defined as a diagnosis agreed upon by 4 out of 8 US pathologists and included 22 biopsies which were negative for dysplasia, 22 biopsies with LGD, 3 biopsies with HGD, 2 biopsies of adenocarcinoma, and 1 biopsy of indefinite for dysplasia. Consensus diagnoses generated by the 8 US GI pathologists were used as a reference to determine the interobserver agreement among the 4 participating pathologists from China.

All 4 participating Chinese pathologists have been working at large medical centers with at least 1500 beds. They have practiced pathology for 16 (2 reviewers) to 20 years (2 reviewers). All 4 reviewers have a strong interest in GI pathology, but none have received GI pathology fellowship training. Three have seen approximately 1000 to 2000 IBD surveillance colonic biopsies, and one has seen more than 2000 IBD surveillance biopsies during their practice. Three were confident in their IBD dysplasia diagnosis, and one thought that he had average IBD dysplasia diagnostic skills. All of them were familiar with the criteria for the diagnosis of IBD-associated neoplasia [6].

Results of the images captured from digitized full slides by 4 participating pathologists are summarized in Table 2. Overall, a diagnosis was agreed upon by all 4 Chinese pathologists in 35 of 50 cases (70%). Among the 22 biopsies which were negative for dysplasia, 21 (95.4%), 20 (90.9%), 20 (90.9%), and 19 (86.4%) cases were concurred by the four reviewers in China, respectively, with one (4.6%), 2 (9.2%), and 1 (4.6%) diagnosed as LGD by reviewers 1, 2, and 3 and 1 (4.6%) and 3 (13.6%) over diagnosed as indefinite for dysplasia by reviewers 3 and 4, respectively. Among those 22 images with a consensus diagnosis of LGD, 16 (72.7%), 20 (90.9%), 21 (95.4%), and 19 (86.4%) cases were concurred by the four reviewers in China as LGD, respectively, with 3 (13.6%%), 1 (4.6%), and 1 (4.6%) diagnosed as HGD by reviewers 1, 2, and 4 and 3 (13.6%) and 1 (4.6%) diagnosed as indefinite for dysplasia by reviewers 1 and 2. One (4.6%) and 2 (9.2%) were diagnosed as negative for dysplasia by reviewers 3 and 4. For 3 cases with a consensus diagnosis of HGD, 3 (100%), 3 (100%), 2 (66.7%), and 2 (66.7%) were concurred as HGD by the four reviewers from China. One (33.3%) was diagnosed as LGD by reviewers 3 and 4. For the 2 cases with a consensus diagnosis of CAC, 2 (100%), 2 (100%), 1 (50%), and 1 (50%) were concurred as CAC. One (50%) was diagnosed as HGD by reviewers 3 and 4. For the case with a consensus diagnosis of indefinite for dysplasia, two reviewers graded as negative, one as LGD, and one as indefinite for dysplasia.

**Figure 1:**
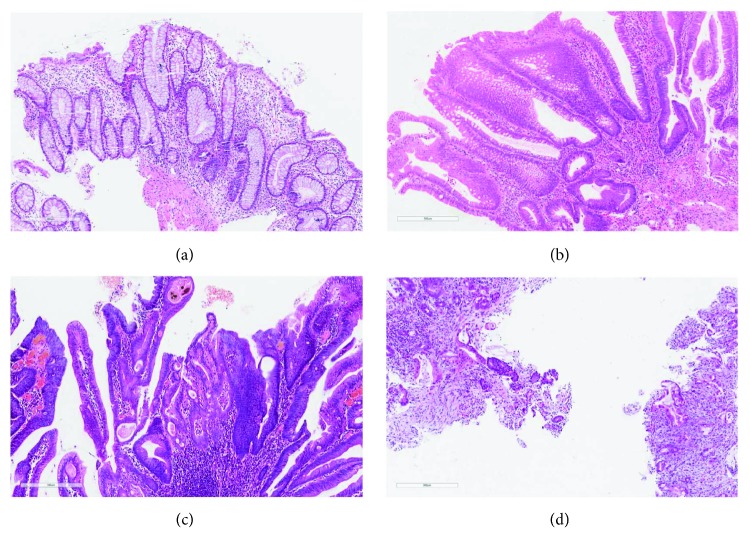
Examples of colonic biopsies with consensus diagnoses by US pathologists were concurred by all 4 Chinese pathologists. (a) There was cryptal distortion, but without nuclear enlargement and hyperchromasia. Surface maturation was present. This case was interpreted as negative for dysplasia (H&E, 200x). (b) This biopsy showed hyperchromatic and enlarged nuclei without surface maturation. The overall features supported a diagnosis of low-grade dysplasia (H&E, 200x). (c) This colonic biopsy showed hyperchromatic nuclei without surface maturation. There was focal nuclear pleomorphism, loss of polarity, and architectural complexity, thus was interpreted as high-grade dysplasia (H&E, 200x). (d) This colonic biopsy showed proliferation of small glands with nuclear pleomorphism and loss of polarity, and desmoplasia, features diagnostic of invasive adenocarcinoma (H&E, 200x).

**Figure 2:**
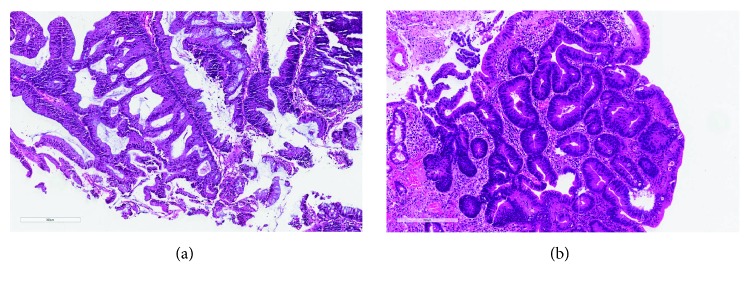
One colonic biopsy with a consensus diagnosis of high-grade dysplasia by US pathologists was read as low-grade dysplasia by two Chinese pathologists (a) (H&E, 200x). The dysplastic glands had very bland nuclear features but a complex cribriform architecture, thus was interpreted as high-grade dysplasia by US pathologists. Another example of colonic biopsy with a consensus diagnosis of low-grade dysplasia by US pathologists was read as high-grade dysplasia by three Chinese pathologists (b) (H&E, 200x). The dysplastic glands had maintained nuclear polarity and without obvious nuclear pleomorphism. The architecture was focally complex with impending cribriform glands, but was regarded within the low-grade dysplasia spectrum by US pathologists.

There was substantial interobserver agreement among 4 pathologists from China on the interpretation of IBD-associated neoplasia (kappa value 0.68, 95% confidence interval 0.56–0.78) (Table 3). Using consensus diagnoses as references, the agreement between each pathologist from China and the consensus diagnosis revealed kappa values ranging from 0.75 to 0.80 (Table 3) indicating substantial agreement between each pathologist and the consensus diagnosis. Colonic biopsies with US consensus diagnosis of negative for dysplasia, LGD, HGD, and cancer agreed by all 4 Chinese pathologists are shown in Figures 1(a)–1(d). One example of a consensus HGD read as LGD by two Chinese pathologists is shown in Figure 2(a). Another example of a consensus LGD read as HGD by three Chinese pathologists is shown in Figure 2(b).

In addition to grading IBD-associated neoplasia, a variety of features (surface maturation, nuclear enlargement, nuclear hyperchromasia, nuclear stratification, and abnormal mitoses) previously reported to be associated with a diagnosis of dysplasia were evaluated by the 4 Chinese participating pathologists (see Table 1). Abnormal mitoses were only rarely observed with a frequency of 10 out of 200 readings (5%) with reviewer 2 reporting these features in 5 cases (2 HGD, 2 cancer, and 1 LGD) and reviewer 4 reporting these features in 5 cases (1 HGD, 2 carcinoma, and 2 LGD). The other two pathologists reported no abnormal mitoses in any of the cases. Due to the low frequency, abnormal mitoses were excluded from the final analysis. The sensitivity, specificity, positive predictive value, and negative predictive value for surface maturation, nuclear enlargement, nuclear hyperchromasia, and nuclear stratification were determined using negative versus other diagnoses (indefinite for dysplasia, LGD, HGD, and carcinoma). The presence of surface maturation had the highest positive predictive value of 93.33%, 100%, 100%, and 96.43% for reviewers 1, 2, 3, and 4, respectively (Table 4). This feature also had a negative predictive value of 100%, 100%, 95.45%, and 90.91% for reviewers 1, 2, 3, and 4, respectively. These results confirm that the presence of surface maturation is associated with a diagnosis of negative for dysplasia. The performance of nuclear enlargement, nuclear hyperchromasia, and nuclear stratification is not as good as the presence of surface maturation (Table 4).

Features previously reported to be associated with HGD diagnosis (nuclear pleomorphism, loss of nuclear polarity, architectural complexity, cribriform glands, and/or papillary configuration) and stratification reaching more than half way of the crypt epithelium thickness [6] were also evaluated by the reviewers. Because papillary configuration was noted at an extremely low frequency (4 of 200 readings, 2%), this feature was removed from the final analysis. The sensitivity, specificity, positive predictive value, and negative predictive value for nuclear pleomorphism, loss of nuclear polarity, nuclear pleomorphism or loss of polarity, cribriform architecture, any of the features (nuclear pleomorphism, loss of nuclear polarity, or cribriform architecture), and stratification reaching more than half way of the crypt epithelium thickness were determined in HGD or cancer and compared with cases with negative for, indefinite for, or LGD. Loss of nuclear polarity showed a positive predictive value for HGD/adenocarcinoma of 100% for all 4 reviewers (Table 5). However, it had a low sensitivity for two reviewers (75% and 83.33% for reviewers 1 and 2). The combination of nuclear pleomorphism, loss of nuclear polarity, or cribriform glands had the highest negative predictive value of 100% for all 4 reviewers (Table 5). These results confirm that, if the biopsy does not have nuclear pleomorphism, loss of polarity, or cribriforming of glands, it should not be diagnosed as HGD or carcinoma.

## 5. Discussion

Recent studies have shown an increasing incidence of UC and CD in Asia including China, estimated to be 0.4 to 2.1 per 100,000 for UC and 1.37 per 100,000 for CD, respectively [2]. UC and Crohn's colitis have been long known risk factors for colorectal cancer [10, 12, 16–19]. In two recent meta-analyses, the overall incidence rate of CAC among 181,923 and 54,478 UC patients was 1.58 to 3 per 1000 patient years, respectively [20, 21]. Although randomized trials to show the effectiveness of surveillance programs in reducing IBD-associated mortality are lacking, many retrospective case series studies have revealed a benefit for surveillance in patients with IBD [12, 22]. For IBD patients entering a surveillance program, the standard of care includes periodic colonoscopic exams with protocol biopsies (4 quadrant biopsies every 10 cm) and targeted biopsies if a lesion is seen to detect dysplasia [5]. The effectiveness of a surveillance program relies on its ability to detect and diagnose early neoplastic lesions, primarily colitis-associated dysplasia, which is the earliest histologic marker predicting neoplastic progression in IBD.

Our study is the first study to examine interobserver agreement among pathologists practicing in China on IBD neoplasia interpretation. Although a few large medical centers have already started subspeciality sign-out in their pathology practice in China, the majority are still using a general sign-out model. So far, there has been no any formal GI pathology fellowship training program in China. Thus, the evaluation of interobserver agreement on IBD neoplasia among pathologists practicing in China and compared their interpretation with US GI pathologists who received GI pathology fellowship training or had strong GI pathology interest that would be ideal to ensure the colonic surveillance biopsies in China will be read adequately. This assurance is important, because for the time being, the management of IBD patients with dysplasia in China is performed according to the guidelines primarily developed in Western countries, including the US. Our study took the advantage of telepathology, a practice of remote diagnostic consultation increasingly used nationally and internationally [23], to determine interobserver agreement on IBD neoplasia interpretation.

Our current study revealed that there is a substantial degree of interobserver agreement on IBD surveillance colonic biopsy interpretation among the 4 participating pathologists in China (kappa value 0.68, 95% confidence interval 0.56–0.78). The agreement of each reviewer with the consensus diagnosis generated was substantial as well (kappa from 0.75 to 0.80). These results are similar to a recent study [11] but is different from some prior, relatively older studies [7–9]. The potential reasons for the discrepancy could be different study periods (cases from 90's to early year 2000 versus current), different patient populations, and glass slides versus captured images.

Histological features previously reported to be associated with dysplasia diagnosis such as lack of surface maturation, nuclear enlargement, nuclear hyperchromasia, and nuclear stratification [6] were reviewed in this study by the Chinese pathologists. Indeed, a lack of surface maturation had the highest positive predictive value as well as a good negative predictive value. These results confirm that the presence of surface maturation supports a diagnosis of negative for dysplasia. Other histologic features such as nuclear enlargement, nuclear hyperchromasia, and nuclear stratification did not perform as well as the assessment of surface maturation. Although the presence of inflammation in the colon harboring IBD-related neoplasia was not well characterized clinically and endoscopically in this study, we found no association of degree of inflammation at the histology level with neoplasia in the biopsies (data not shown). This might reflect the fact that the cumulative burden of inflammation in IBD rather than the status of inflammation at diagnosis is associated with the risk of neoplasia.

Histological features previously reported for HGD such as nuclear pleomorphism, loss of nuclear polarity, architectural complexity (cribriform glands and/or papillary configuration), and stratification reaching more than half way of the crypt epithelium thickness [6] were also evaluated by the Chinese reviewers in this study. The sensitivity, specificity, positive predictive value, and negative predictive value of these features and two combinations of these features (nuclear pleomorphism or loss of nuclear polarity; nuclear pleomorphism, loss of nuclear polarity, or cribriform glands) were determined for the diagnosis of HGD or carcinoma. Our results revealed loss of nuclear polarity with an excellent positive predictive value for HGD or carcinoma. However, it had a low sensitivity for two reviewers (75% and 83.33% for reviewers 1 and 2). The assessment of nuclear pleomorphism and cribriform architecture is not as reliable as loss of nuclear polarity for a diagnosis of HGD, perhaps related to different thresholds for recognizing cribriform glands as cribriform. If cribriform architecture is defined as gland-in-gland configuration, the example in Figure 2(a) should be recognized as HGD while the example in Figure 2(b) may represent a borderline lesion, causing diagnostic discrepancy between LGD and HGD. Our results suggest that loss of nuclear polarity is the most reliable feature of HGD and cancer and cases with architectural complexity such as cribriforming should be diagnosed as HGD even if there is nuclear uniformity and maintained nuclear polarity. The combination of nuclear pleomorphism, loss of nuclear polarity, or cribriform glands had the highest negative predictive value of 100% for all 4 reviewers. These results confirm that, if a colonic biopsy does not have any of these features, that is, nuclear pleomorphism, loss of polarity, or cribriforming gland, it should not be diagnosed as HGD or carcinoma.

There are several strengths of this study. First, this is the first study to examine the interobserver agreement among Chinese pathologists for IBD-associated neoplasia diagnosis. Second, the performance of these Chinese pathologists was compared to the consensus diagnosis rendered by 8 GI pathologists who either received GI pathology fellowship training or had extensive experience in GI pathology by practicing in hospitals with a large volume of IBD surveillance colonic biopsies. Third, the study used the newly matured technology, telepathology. More specifically, the substantial interobserver agreement for the interpretation of colonic neoplasia in IBD using digitized images among Chinese pathologists as well as between each Chinese pathologist and a consensus diagnosis generated by US GI pathologists as revealed in this study supports the potential use of telepathology in facilitating access to pathology expertise, either in China or international, to improve the diagnosis and clinical management of IBD-associated neoplasia when and where local expertise is not available.

This study has several imitations. First, it used images captured from digitized full-slide images and some of the slides had faded staining. Second, the number of cases was small and no intraobserver agreement study was performed in this study. For example, the small number of HGD cases in this study may not be sufficient in representing the different patterns of HGD which may be encountered in clinical practice. Third, the biopsies were from patients with a clinical diagnosis of IBD in China without having a histological confirmation of their IBD by the study pathologists. Fourth, the endoscopic appearance of the colon where these biopsies were taken and the clinical outcome of the patients were not available. Last but not the least, the participating pathologists in this study were from large IBD centers in China; thus, the results from this study may not be applicable to the pathologists practicing in smaller hospitals where IBD colonic surveillance biopsies are not as commonly encountered.

In summary, this study revealed a substantial interobserver agreement among Chinese pathologists for IBD-associated neoplasia diagnosis. Each Chinese pathologist also had a substantial agreement with a consensus diagnosis rendered by 8 US GI pathologists. This study confirmed the reliability of surface maturation for the diagnosis of negative for dysplasia. This study also confirmed that if the biopsy does not have nuclear pleomorphism, loss of polarity, or cribriforming glands, it should not be diagnosed as HGD or carcinoma. Additional studies using a large number of histologically confirmed IBD surveillance colonic biopsies would be helpful to support these findings in the future.

## Figures and Tables

**Table 1:** Features evaluated in this study.

Features	Definition	Score/code
*Surface maturation*		
Absent	The size and staining quality of surface and crypt basal nucleus are identical.	0
Present	The surface nucleus is smaller and stains paler than the crypt basal nucleus.	1
*Nuclear enlargement*		
Absent	The nucleus of interest is of normal size or similar to nearby nonneoplastic epithelial nucleus.	0
Present	The nucleus of interest is larger than nearby nonneoplastic epithelial nucleus.	1
*Nuclear hyperchromasia*		
Absent	The nucleus of interest has similar staining quality similar to adjacent nonneoplastic epithelial nucleus.	0
Present	The nucleus of interest stains darker than normal or darker than adjacent nonneoplastic epithelial nucleus.	1
*Nuclear stratification*		
Absent	The nucleus is not overlapping with each other, and there is a single layer of nuclei in the glandular lining epithelium.	0
Present, only involving the basal half of the crypt epithelium	The nucleus shows overlapping with each other, more than single layer of nuclei in the glandular lining epithelium, but occupying the basal half of the glandular epithelium thickness.	1
Present, reaching more than half way to the crypt epithelium	The nucleus shows overlapping with each other, more than single layer of nuclei in the glandular lining epithelium, and reaching more than half way to the glandular epithelium thickness.	2
*Nuclear pleomorphism*		
Absent	The nucleus of interest has uniform size and shape.	0
Present	The nucleus of interest has different size and shape.	1
*Loss of nuclear polarity*		
Absent	The long axis of the nucleus is perpendicular to the basement membrane and arranged paralleling to each other.	0
Present	The long axis of the nucleus is no longer perpendicular to the basement membrane and arranged in a haphazard way with each other.	1
*Architectural complexity*		
Absent	Small straight glands.	0
Present, cribriform	Gland-in-gland.	1
Present, papillary	Papillary structure on the surface and/or in the lumen of the glands.	2
Present, cribriform and papillary	Both cribriform and papillary structures present.	3
*Abnormal mitosis*		
Absent	Only normal mitosis seen.	0
Present	Mitosis with three or more poles.	1

**Table 2:** Interpretation of IBD colonic biopsies by four pathologists from China in comparison to the consensus diagnosis from US.

	Consensus negative (*N* = 22)	Consensus LGD (*N* = 22)	Consensus HGD (*N* = 3)	Consensus carcinoma (*N* = 2)	Indefinite for dysplasia (*N* = 1)
Reviewer 1	21 (negative) 1 (LGD)	16 (LGD) 3 (IND) 3 (HGD)	3 (HGD)	2 (carcinoma)	1 (negative)

Reviewer 2	20 (negative) 2 (LGD)	20 (LGD) 1 (IND) 1 (HGD)	3 (HGD)	2 (carcinoma)	1 (negative)

Reviewer 3	20 (negative) 1 (IND) 1 (LGD)	21 (LGD) 1 (negative)	2 (HGD) 1 (LGD)	1 (carcinoma) 1 (HGD)	1 (LGD)

Reviewer 4	19 (negative) 3 (IND)	19 (LGD) 2 (negative) 1 (HGD)	2 (HGD) 1 (LGD)	1 (carcinoma) 1 (HGD)	1 (IND)

Note: LGD: low-grade dysplasia; HGD: high-grade dysplasia; IND: indefinite for dysplasia. US consensus is defined as diagnostic agreement by at least 4 participating US GI pathologists.

**Table 3:** Interobserver agreement between each reviewer from China and consensus diagnosis rendered by 8 US GI pathologists.

Reviewer	Pathology experience (year)	Kappa between review pathology and the consensus diagnosis [95% confidence interval]	Agreement	*P* value
1	16	0.75 [0.58, 0.90]	Substantial	<0.05
2	20	0.80 [0.64, 0.94]	Substantial	<0.05
3	16	0.80 [0.65, 0.93]	Substantial	<0.05
4	20	0.75 [0.56, 0.78]	Substantial	<0.05

**Table 4:** Diagnostic use of each feature in the diagnosis of colitis-associated dysplasia (CAD) (negative versus other).

Features in the diagnosis of CAD	Negative versus other			
	Sen (%)	Spe (%)	PPV (%)	NPV (%)
*Surface maturation*				
Reviewer 1	100	93.33	93.33	100
Reviewer 2	100	100	100	100
Reviewer 3	96.55	100	100	95.45
Reviewer 4	93.10	95.24	96.43	90.91
*Nuclear enlargement*				
Reviewer 1	100	31.82	65.12	100
Reviewer 2	89.66	76.19	83.87	84.21
Reviewer 3	96.55	66.67	80	93.33
Reviewer 4	100	61.90	78.38	100
*Hyperchromasia*				
Reviewer 1	92.86	40.91	66.67	81.82
Reviewer 2	93.10	42.86	69.23	81.82
Reviewer 3	96.55	66.67	80.00	93.33
Reviewer 4	100	52.38	74.36	100
*Nuclear stratification*				
Reviewer 1	96.43	90.91	93.10	95.24
Reviewer 2	96.55	47.62	71.79	90.91
Reviewer 3	100	71.43	82.86	100
Reviewer 4	100	71.43	82.86	100

**Table 5:** Diagnostic use of each feature in the diagnosis of colitis-associated high-grade dysplasia HGD or carcinoma.

Features in the diagnosis of colitis-associated HGD or carcinoma	HGD and carcinoma versus others			
	Sen (%)	Spe (%)	PPV (%)	NPV (%)
*Nuclear pleomorphism*				
Reviewer 1	75	100	100	95.45
Reviewer 2	50	97.73	75	93.48
Reviewer 3	100	100	100	100
Reviewer 4	100	100	100	100
*Loss of polarity*				
Reviewer 1	75	100	100	95.45
Reviewer 2	83.33	100	100	97.78
Reviewer 3	100	100	100	100
Reviewer 4	100	100	100	100
*Nuclear pleomorphism or loss of polarity*				
Reviewer 1	75	100	100	95.45
Reviewer 2	83.33	97.73	83.33	97.73
Reviewer 3	100	100	100	100
Reviewer 4	100	100	100	100
*Cribriform architecture*				
Reviewer 1	62.5	100	100	93.33
Reviewer 2	100	100	100	100
Reviewer 3	100	97.83	80.00	100
Reviewer 4	60	95.56	60	95.56
*Nuclear pleomorphism or loss of polarity or cribriform*				
Reviewer 1	100	100	100	100
Reviewer 2	100	97.73	85.71	100
Reviewer 3	100	100	100	100
Reviewer 4	100	84.85	71.43	100
*Nuclear stratification reaching more than half way of the crypt epithelium*				
Reviewer 1	100	100	100	100
Reviewer 2	83.33	97.73	83.33	97.73
Reviewer 3	100	100	100	100
Reviewer 4	100	95.56	71.43	100
